# New Society

**Published:** 1882-04

**Authors:** 


### NEW SOCIETY.

A number of veterinarians met in Dublin, and formed a new society, to be called the Irish Central Veterinary Medical Society, electing T. D., Lambert, F.R.C.V.S., President; A. E. Ross, M.R.C.V.S., T. H. Suncocks, M.R.C.V.S., John Malcolm, M.R.C.V.S., Vice-Presidents; J. J. Sperring M.R.C.V.S., Secretary and Treasurer.

